# Design of news recommendation model based on sub-attention news encoder

**DOI:** 10.7717/peerj-cs.1246

**Published:** 2023-03-09

**Authors:** Wenting Zhang

**Affiliations:** College of Art and Design, Xi’an Mingde Institute of Technology, Xi’an, Shaanxi, China

**Keywords:** News recommendation, Sub-attention, CNN, Multi-head self-attention mechanism

## Abstract

To extract finer-grained segment features from news and represent users accurately and exhaustively, this article develops a news recommendation (NR) model based on a sub-attention news encoder. First, by using convolutional neural network (CNN) and sub-attention mechanism, this model extracts a rich feature matrix from the news text. Then, from the perspective of image position and channel, the granular image data is retrieved. Next, the user’s news browsing history is injected with a multi-head self-attention mechanism, and time series prediction is applied to the user’s interests. Finally, the experimental results show that the proposed model performs well on the indicators: mean reciprocal rank (MRR), Normalized Discounted Cumulative Gain (NDCG) and area under the curve (AUC), with an average increase of 4.18%, 5.63% and 6.55%, respectively. The comparative results demonstrate that the model performs best on a variety of datasets and has fastest convergence speed in all cases. The proposed model may provide guidance for the design of the news recommendation system in the future.

## Introduction

Due to the sheer quantity of content available, there is a risk of information overload when using online news platforms (such as Google News). Recommending relevant news based on a user’s interests is an excellent way to reduce information overload. In contrast to other types of projects such as movies, books, educational resources, etc., news typically has a short shelf life and is frequently replaced by newer news. Some users’ interests may be long-lasting, whereas others may be triggered by particular contexts or temporary needs, and the rate at which these interests change is typically less stable than in other fields (Ge et al., 2020). However, conventional collaborative filtering techniques have difficulty keeping up with the ever-changing interests of users because they do not account for the sequential browsing data of users (Wu et al., 2020).

Consequently, a number of researchers have proposed serialized news recommendation (NR) methods, which typically use recurrent neural network (RNN) and attention mechanism (AM) to model users’ historical interaction behaviors and capture users’ sequential reading patterns. The RNN uses the session information from the user’s browsing history as input sequence, the gated recurrent unit (GRU) captures the sequence information from the user’s behavior sequence to learn the user’s long-term interest, and the final hidden state of the GRU network is used as output to create a user representation (Santosh, Saha & Ganguly, 2020). The recommendations made to users now take their immediate and long-term objectives into account. Some researchers have turned to AM in order to forecast future preferences based on past actions (Lee et al., 2020).

Various methods for recommending news articles based on their subject matter have been proposed. Some researchers have suggested that autoencoders be used to encode articles and RNNs to encode end users  (Zhang, Jia & Wang, 2021). According to the comparative analysis of these models, the following issues frequently arise in the current NR system. The first challenge is determining how to efficiently extract news information from news content, which is essential for NR, especially when processing lengthy news stories and capturing key words in the text to reduce the effect of irrelevant words on semantics (Tran, Hamad & Zaib, 2021). The second issue is how to simulate user preferences accurately. Current recommender systems frequently omit user-related item features from their representations when building user profiles.

To address the aforementioned issues, this article proposes a NR model based on a sub-attention news encoder which helps the neural network extract news features more efficiently and accurately by emphasizing relevant information and suppressing irrelevant information. The proposed system includes both the news encoding module (NE) and the user preference encoding module (UE). The following is a summary of the article’s major contributions:

(1) This article first incorporates a sub-attention mechanism (SAM), then learns key texts with convolutional neural network (CNN), and finally fuses the contextual features of news texts to extract more effective news features.

(2) This article first models old news records using a GRU network to extract sequence characteristics and then models the characteristics of browsing content. In addition, the article employs fused multi-head AM to model the characteristics of user browsing history. Finally, an attention mechanism is used to learn user preference representations from previous news features.

## Related Work

**Figure 1:**
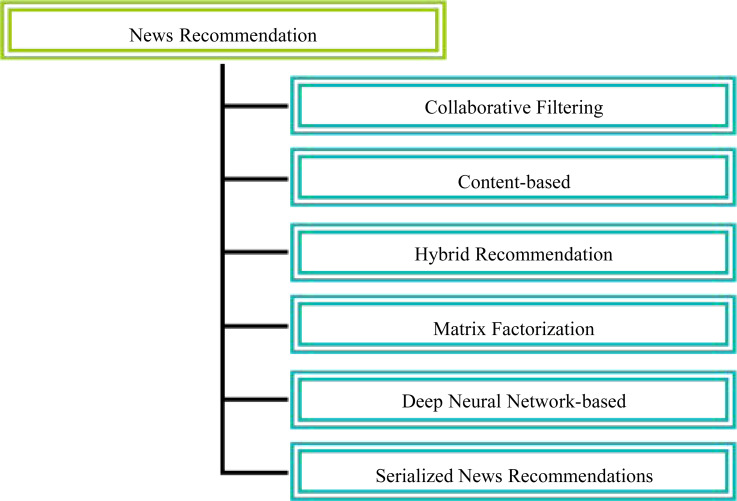
Classification of news recommendation methods. Among the classic techniques are collaborative filtering, content, and hybrid approaches, among others. By analyzing the ratings that users have given for similar items or for ratings given by other users, collaborative filtering-based approaches attempt to predict the preferences of users.

The classification of NR methods is shown in Fig. 1.

Among the classic techniques are collaborative filtering, content, and hybrid approaches, among others. By analyzing the ratings that users have given for similar items or for ratings given by other users, collaborative filtering-based approaches attempt to predict the preferences of users. For instance, in order to inform their recommendations, scientists consider both explicit and implicit ratings provided by users. However, when these strategies are first implemented, they frequently encounter difficulties due to a ”cold start”, which occurs because news articles are frequently replaced  (Ge et al., 2020; Wu et al., 2020; Lee et al., 2020; Zhang, Jia & Wang, 2021).

By analyzing the content of a user’s news-browsing habits, content-based methods can mitigate the cold-start problem and provide relevant article recommendations. These techniques are known as “content-based” techniques. Some academics have proposed a content-based deep neural network model that employs cosine similarity calculations to determine the degree to which two documents are associated (Tran, Hamad & Zaib, 2021; Khattar, Kumar & Varma, 2018; Wang, Wang & Lu, 2022). Content-based recommendation, which is based on deep learning and is an explicit modeling of news features and user interest features, possesses strong interpretability and has been vigorously and successfully developed. In addition, content-based recommendation is highly interpretable and has been actively and successfully developed. Researchers from 2017 proposed combining a denoising autoencoder for news representation learning and a GRU network for news-based user representation learning. Researchers have proposed a number of multi-head AM-based NR models in 2019 (Zhang, Wang & Ren, 2022). The primary purpose of these models is to extract the relationship between context and news by employing multi-head AM at both the word and news levels (Tran et al., 2022). Some of the researchers also combed CNN for news features, which were extracted from the source and subjected to a subject-based classification. The extraction of features yielded extremely satisfying results (Qian, Wang & Hu, 2021). Some researchers employs a recurrent neural network and an id embedding to determine the user’s long-term and short-term interests. There are also problems with suggestions based on the displayed content. There is a gap in the construction of the portrait because the majority of approaches rely on article-level matching, which has the potential to conceal semantic and interest features buried in finer-grained news segments (Ma & Zhu, 2019; Shi, Ma & Wang, 2021).

In most instances, a hybrid approach consolidates distinct recommendation procedures into a single overarching strategy. A few researchers have proposed a two-stage recommendation framework that employs both collaborative filtering and content-based approaches. Because these methods disregard the sequential information in a user’s browsing history, it is challenging to determine how users’ interests evolve over time (Wu, Wu & Qi, 2021; Qi et al., 2021).

In NR, modeling the news accurately is a crucial responsibility. Some works model and obtain semantic representations of news utilizing only a single DL technique. CNN’s algorithm is widely employed to extract news text features. Several academics, for instance, have proposed a DL-based NR model (Shi, Xu & Hu, 2021). The content representation module of this system performs convolution calculations beginning at the word level on the text content of CNN news stories in order to generate the embedded representation of news content (Ke, 2020). In the DAINN model, CNN is used to represent the word-level content of news text content. This is one of the many applications CNN offers (Wang et al., 2021). Some works select multiple types of news data to model the news in order to enrich the information with semantically relevant content  (Qiu, Hu & Wu, 2022). For news editors, researchers have developed a professional news screening and recommendation system. This system aims to solve the issue of ambiguous news screening standards, which arise when news editors place more emphasis on the quality of the news text and less emphasis on metadata such as keywords and topics when screening news. The researchers propose modeling news and predicting its screening criteria using two distinct data types. These records contain the text and category of news articles (Sun et al., 2021; Halder, Poddar & Kan, 2018). A CNN model with one convolution layer and a total of 1,050 convolution kernels is used to represent textual content in this framework by capturing latent semantic patterns in word sequences, while one-hot vectors are used to represent elements such as news categories (Wu, Wu & Wang, 2021). The CNN model contains a total of 1050 convolution kernels. After collecting data and information, the final step is to combine the two to forecast the likelihood of news screening. In addition, the model constructs CNN based on characters, which increases its generalizability across multiple languages. Since there is insufficient semantic information in the characters, the news semantic features extracted by character-level CNN will not be sufficiently rich, and the expansion of input sequences may increase computational costs (Vinh Tran, Nguyen Pham & Tay, 2019).

Some scholars propose the DAN model, which integrates news summaries, which are more informative than news headlines, into news data  (Zan, Zhang & Meng, 2021). This is done while considering news headlines. DAN is based on the idea of two concurrent CNNs so that users can comprehend the news feature representation. The pulse coupled neural network (PCNN) receives headlines and summaries at the word level as inputs, learns representations of news at the level of headlines and summaries, and combines these representations to form the final news feature representation (Wang, Dai & Cao, 2023). PCNN-based models are more competitive than models that rely solely on news headlines because they are supported by a greater number of data features. Previous research has demonstrated that CNN is widely used for modeling news; however, due to CNN’s fixed receptive field, it is not suitable for modeling longer news word sequences. This limitation eliminates CNN as a suitable tool for this task (Wang, Zhang & Rao, 2020; Deng, Li & Liu, 2021).

Using matrix factorization as a foundation, social information has been incorporated into the recommendation model in order to capture more expressive user preference vectors. characterized by a high degree of adaptability and an enhanced capacity for recommendation. The two subcategories of recommendation models that fall under the category of models that use matrix factorization are collaborative factorization methods and social regularization methods (Xia, Huang & Xu, 2020). Due to the presence of the user feature vector in both the user-item rating matrix and the social matrix, the collaborative decomposition method can decompose both of these matrices simultaneously. The SOREC-based model is an example of a collaborative decomposition technique based on matrix factorization (Wang, Xi & Huang, 2020). This method begins by decomposing the user-item rating matrix and the social relationship matrix into a vector of latent user features. Decomposing the user-item rating matrix and limiting the user feature vector with the social matrix is a crucial aspect of the matrix factorization-based social regularization method. Several researchers have proposed a model of matrix factorization with social regularization. Utilizing the social matrix, this model regularizes the user eigenvectors.

Specialists have proposed DNN-based nonlinear recommendation methods as an alternative to matrix factorization-based tasks due to their limitations. Then, DNN is used to extract user nonlinear feature vectors from the social relations matrix and incorporate them into the factorization of the probability matrix for scoring prediction. Social relations are initially input into a graph embedding model in order to pre-train user node embeddings. DeepSOR is the name of the model in question (Jiang, Wang & Wei, 2020). DSCF, an additional DNN-based social recommendation model, can utilize high-order social neighbor information to increase the amount of social information and user-item interaction data it can extract (Chen, Cao & Chen, 2021).

Sessions are frequently mentioned throughout the serialization recommendations, and sequences are constructed from sessions. Recent research heavily relies on RNN modeling for a variety of purposes. Using tensor-based CNN and RNN to record the click-order of users’ selected stories, researchers have successfully extracted session-level representations of news features (Wu, Wu & Qi, 2021; Ke, 2020). The most recent hidden state of the GRU network can be used to infer the user’s short-term interests. Following its widespread adoption in other machine learning applications, supervised learning has grown in popularity in recent years, resulting in a rise in the use of AI for recommendation tasks. Some academics have only recently begun exploring the viability of using AM to survey user preferences. According to Reference Deng, Li & Liu (2021), the purpose of constructing an AM-based RNN was to record the order in which users clicked on various news articles. Google initially proposed SAM, and the company was the first to implement it for MT. It is possible to compute the SAM representation from the sequence alone, making it a subset of the more general AM. SAM is able to identify long-distance dependencies between words because, unlike conventional AM, it places a greater emphasis on the interactive learning that occurs between multiple words within a sentence (Qiu, Hu & Wu, 2022; Vinh Tran, Nguyen Pham & Tay, 2019; Wang, Xi & Huang, 2020). Academics have proposed a time-sensitive SAM that can learn users’ short-term interests based on their recent browsing sequences and combine these interests with users’ long-term interests to provide more accurate news recommendations. A sequential knowledge-aware recommendation model was the brainchild of a different researcher. This model uses SAM to identify sequential patterns in user interaction logs in order to generate recommendations (Guo, Yin & Chen, 2021).

According to the comparative analysis of these models, the following issues frequently arise in the current NR system. The first challenge is determining how to efficiently extract news information from news content, which is essential for NR, especially when processing lengthy news stories and capturing key words in the text to reduce the effect of irrelevant words on semantics. The second issue is how to simulate user preferences accurately. Current recommender systems frequently omit user-related item features from their representations when building user profiles.

## Method

**Figure 2:**
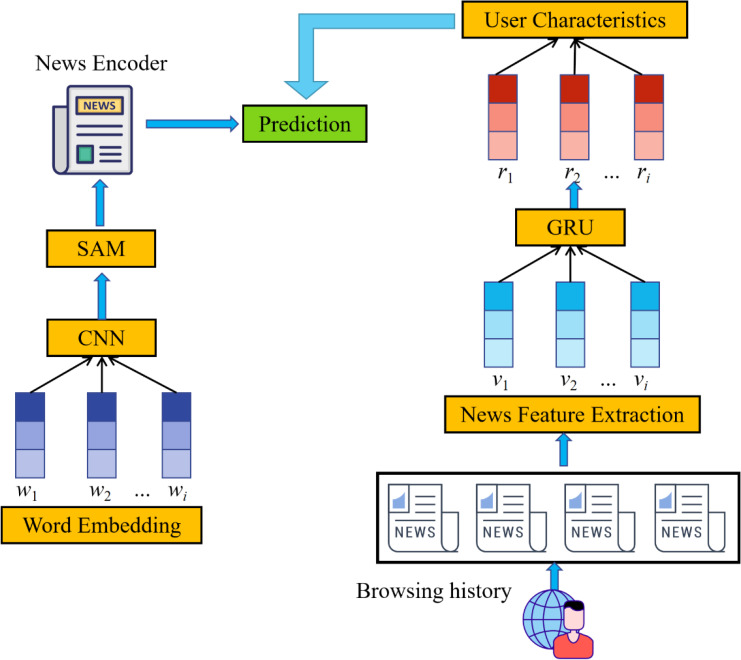
Structure of our method. Initially, a CNN- and SAM-based news feature extraction module will be introduced. Through the utilization of convolutional neural networks (CNNs) and word-level support vector machine (SAM) modeling, this article is able to process lengthy news articles by giving more weight to specific words.

In this article, we build a news recommendation system based on the concept of sub-attention, which allows it to independently learn user features (as shown in Fig. 2). CNN makes use of a text feature extraction model that is based on SAM in order to discover characteristics of users who read the news and possible candidates for news stories. GRU’s time series feature extraction mechanism is able to collect information about the user because it combines the user feature model with the SAM and the MAM. The resulting candidate news prediction ranking model uses both the user’s profile and their browsing history to assign scores to individual articles.

Initially, a CNN- and SAM-based news feature extraction module will be introduced. Through the utilization of convolutional neural networks (CNNs) and word-level support vector machine (SAM) modeling, this article is able to process lengthy news articles by giving more weight to specific words. These techniques enable the efficient extraction of the text’s context, local characteristics, and global characteristics.

News text and its vector representation as

(1) \begin{eqnarray*}& wor{d}_{1},wor{d}_{2},\ldots ,wor{d}_{l}\end{eqnarray*}

(2) \begin{eqnarray*}& Wo= \left( wor{d}_{1},wor{d}_{2},\ldots ,wor{d}_{l} \right) .\end{eqnarray*}

Suppose the number of convolution kernels is *p*, the context-related word vector matrix output by the CNN layer is

(3) \begin{eqnarray*}Co= \left( c{o}_{1},c{o}_{2},\ldots ,c{o}_{p} \right) .\end{eqnarray*}

When the length of the news article is excessive, the semantic vector may not be able to accurately represent all of the sequence’s information. This is due to the fact that the significance of the information carried by the content entered first is easily diminished by subsequent content. We introduce SAM into news text in this article. This helps the neural network extract news features more efficiently and accurately by emphasizing relevant information and suppressing irrelevant information.

Suppose the *i*-th word weight is *w*_*i*_

(4) \begin{eqnarray*}{w}_{i}={q}_{i}\tanh \nolimits \left( w{w}_{i}\ast c{o}_{i}+{b}_{i} \right) \end{eqnarray*}

where *ww*_*i*_ isthe training weight matrix, *b*_*i*_ is the random error. When the activation function changes, *w*_*i*_ is calculated as

(5) \begin{eqnarray*}{w}_{i}= \frac{\exp \nolimits \left( {w}_{i} \right) }{\sum \exp \nolimits \left( {w}_{i} \right) .} \end{eqnarray*}

Further, output the news feature vector *w*_*news*_

(6) \begin{eqnarray*}{w}_{news}=\sum {w}_{i}c{o}_{i}.\end{eqnarray*}

This article develops a user feature extraction model in order to handle the time series features that are introduced by the user’s browsing time. Additionally, in order to extract the correlation between historical news and the key news that affects users, the model must handle the time series features. The first component is a neural network–based GRU time series prediction module. This module outputs the feature matrix *via* the browsing record list in order to take into account the length of time it takes to complete a browsing session.

The historical news matrix after encoding representation is

(7) \begin{eqnarray*}{\mathbf{w}}_{news}= \left[ {w}_{news1},{w}_{news2},\ldots ,{w}_{newsn} \right] .\end{eqnarray*}

The calculation process in the middle of GRU are as follows:

(8) \begin{eqnarray*}{r}_{t}& =\text{sigmod} \left( {W}_{r} \left[ {h}_{t-1},{w}_{newst} \right] \right) \end{eqnarray*}

(9) \begin{eqnarray*}{z}_{t}& =\text{sigmod} \left( {W}_{z} \left[ {h}_{t-1},{w}_{newst} \right] \right) \end{eqnarray*}

(10) \begin{eqnarray*}{h}_{t}& =\tanh \nolimits \left( {W}_{h} \left[ {h}_{t-1},{w}_{newst} \right] \right) \end{eqnarray*}

(11) \begin{eqnarray*}{h}_{t}& ={h}_{t}+{z}_{t}\odot {h}_{t}-{z}_{t}\odot {h}_{t}\end{eqnarray*}

where *W*_*r*_, *W*_*z*_, *W*_*h*_ is the parameter matrix of the corresponding layer, *r*_*t*_ is the reset gate, *z*_*t*_ is the update gate, *h*_*t*_ is the candidate hidden state of *t*, and *h*_*t*_ is the hidden state of *t*.

**Figure 3:**
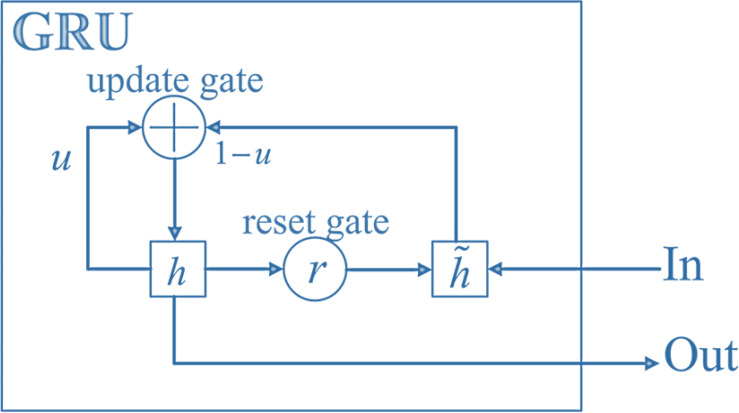
Structure of GRU. In the structure of GRU, the reset gate determines how the new input information is combined with the previous memory, and the update gate defines the amount of previous memory saved to the current time step; reset gates are used to control the degree to which state information from the previous moment is ignored.

The structure of GRU is shown in Fig. 3. In the structure of GRU, the reset gate determines how the new input information is combined with the previous memory, and the update gate defines the amount of previous memory saved to the current time step; Reset gates are used to control the degree to which state information from the previous moment is ignored.

By observing how frequently the same user reads similar articles in the news, MSA (multi-head self-attention) mechanism is able to glean more nuanced characteristics about them. Multiple zoom-click SA units are stacked to create the MSA. Each news item in the input newsgroup must perform an attention calculation with every other news item; this is done so that the system can learn the connection between stories that have been viewed by the same user.

Assuming that the feature matrix of news is *F*, the MSA is calculated as follows:

(14) \begin{eqnarray*}Q& ={W}_{q}\ast F\end{eqnarray*}

(15) \begin{eqnarray*}K& ={W}_{k}\ast F\end{eqnarray*}

(16) \begin{eqnarray*}V& ={W}_{v}\ast F.\end{eqnarray*}

When performing MSA, Q, K, and V are linearly transformed before being input to the scaled dot-product attention, and the parameter W of the linear transformation of Q, K, and V varies from iteration to iteration. This ensures that the linear transformation of Q, K, and V produces the most accurate results possible. The value that is arrived at by applying a linear transformation to the h-scaled dot-product of attention is what is considered to be the end result of the MSA.

**Figure 4:**
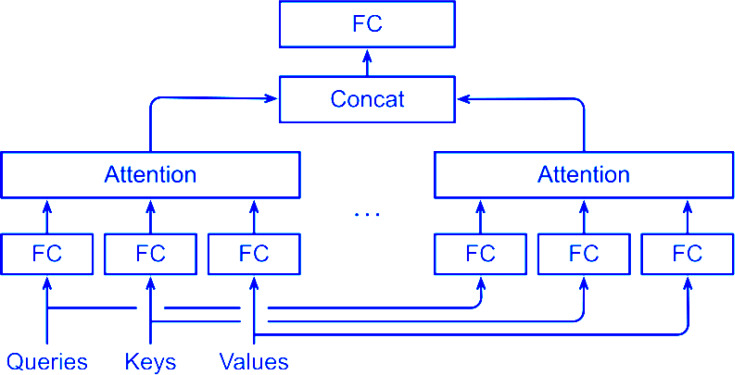
Framework of MSA. The framework of MSA is shown, where FC is a fully connected layer.

The framework of MSA is shown in Fig. 4, where FC is fully connected layer.

The rest of the parameter settings and loss functions in this article are the same as in Reference 14.

## Result

Four datasets taken from the real world are chosen in order to test how well the model that has been proposed works. The first dataset is a news network dataset called CCX, and the second dataset is called Microsoft News Dataset (MIND). The third and fourth datasets both come from Adressa, but the third dataset, known as Adressa1, contains weekly data. The Adressa2 dataset, which comes in at number four, is a monthly dataset. For the purpose of providing adequate historical session information, non-anonymous users with subscription services were chosen, sessions with fewer than three interactions were discarded, and sessions with more than four interactions were kept. The proportional breakdown of the dataset into its training set, validation set, and test set is 8 to 1 to 1. The details of the four datasets are shown in Table 1.

The evaluation indicators used in this article are mean reciprocal rank (MRR), Normalized Discounted Cumulative Gain (NDCG) and area under the curve (AUC).

The average of the reciprocal ranks of the items that were correctly recommended is known as the mean reciprocal rank (MRR). When the rank number is greater than K, the reciprocal rank is equal to 0. The MRR metric considers the ranking order of recommendations; a higher MRR value indicates that the recommended item is more likely to be correct. nDCG is the normalized discounted cumulative gain. When comparing two samples, AUC indicates the likelihood that the positive sample’s predicted value is higher than the negative sample’s. The calculation formulas of the three indicators are as follows:

(17) \begin{eqnarray*}MRR& = \frac{1}{K} \sum _{i=1}^{K} \frac{1}{rank \left( i \right) } \end{eqnarray*}

(18) \begin{eqnarray*}nDCG& = \frac{\sum _{i=1}^{p+q} \frac{I}{\ln \nolimits \left( i+1 \right) } }{\sum _{i=1}^{p} \frac{I}{\ln \nolimits \left( i+1 \right) } } \end{eqnarray*}

(19) \begin{eqnarray*}AUC& = \frac{U-P \left( P+1 \right) }{2PQ} \end{eqnarray*}

where *U* is the ranking of all samples, *P* and *Q* are the number of positive and negative samples.

The specific parameter settings are shown in Table 2.

**Figure 5:**
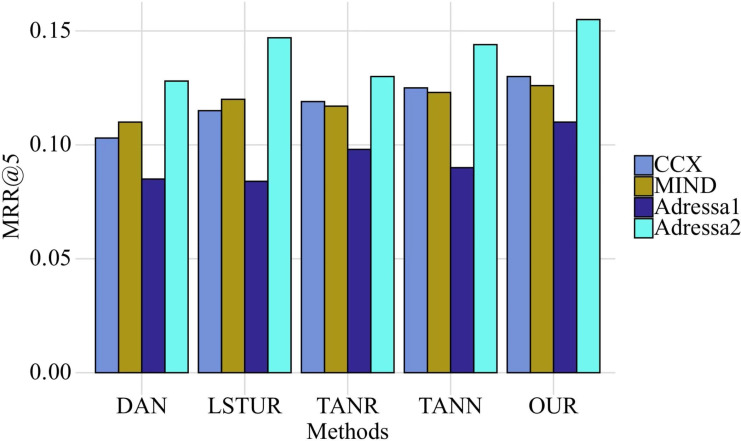
Comparison of MRR@5 of five methods on four datasets. Comparison algorithm This article adopts DAN, LSTUR, TANR and TANN in Khattar, Kumar & Varma (2018); Ma & Zhu (2019); Wu, Wu & Qi (2021); Qi et al. (2021). First, we compare the performance of different datasets in MRR@5 and MRR@15 on four datasets.

**Figure 6:**
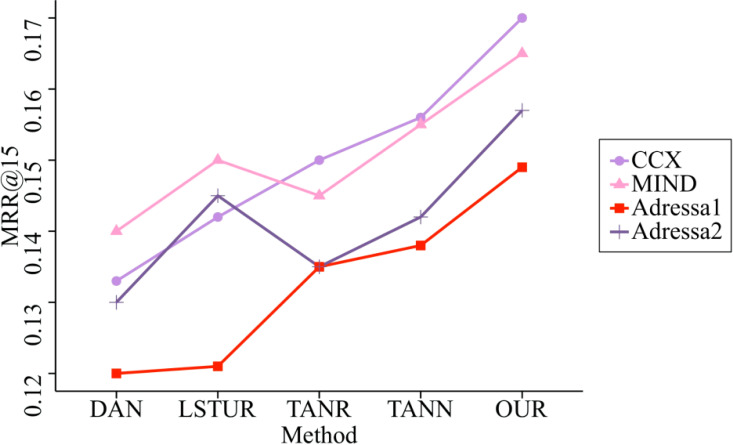
Comparison of MRR@15 of five methods on four datasets. The improvement interval for the MRR@5 model is between 0.39% and 5.56%, and the average improvement rate of the MRR@5 model is 3.85%. This rate is compared to the comparison model.

Comparison algorithm This article adopts DAN, LSTUR, TANR and TANN in Khattar, Kumar & Varma (2018); Ma & Zhu (2019); Wu, Wu & Qi (2021); Qi et al. (2021). First, we compare the performance of different datasets in MRR@5 and MRR@15 on four datasets, as shown in the Figs. 5 and 6.

It is clear from looking at Figs. 5 and 6 that, out of the four different data sets, the indicators produced by this model are the most accurate ones. The improvement interval for the MRR@5 model is between 0.39% and 5.56%, and the average improvement rate of the MRR@5 model is 3.85%. This rate is compared to the comparison model. The improvement range goes from 0.95% to 5.78%, with the average improvement rate for MRR@15 coming in at 4.18%.

**Figure 7:**
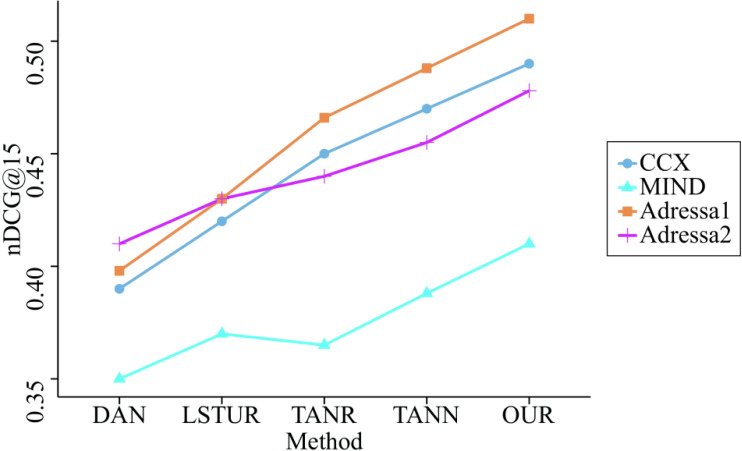
Comparison of nDCG@15 of five methods on four datasets. This figure and Fig. 8 reveal that our method’s indicators have the highest overall performance of all the benchmark methods. They have demonstrated an average increase in nDCG@15 and AUC of 5.63% and 6.55%, respectively, proving that the model presented in this article is accurate.

**Figure 8:**
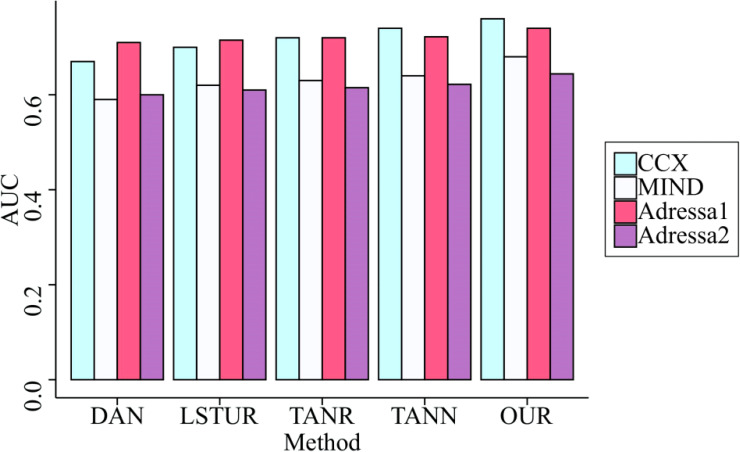
Comparison of AUC of five methods on four datasets. GRU is used to collect the information that users provide sequentially. In addition, unlike other benchmark methods, the proposed model not only considers the user interaction behavior that occurs within a session, but also the correlation of user interests that occurs between sessions; as a result, the performance of the recommendation has been enhanced even further.

Further, we compared the remaining two indicators on the four datasets, and the comparison results are shown in Figs. 7 and 8.

**Table 1:** Detail of four datasets. The first dataset is a news network dataset called CCX, and the second dataset is called the Microsoft News Dataset (MIND). The third and fourth datasets both come from Adressa, but the third dataset, known as Adressa1, contains weekly data. The fourth datasbet, the Adressa2 dataset, is a monthly dataset.

	CCX	MIND	Adressa1	Adressa2
News number	120375	176550	173525	695135
User number	9549	10000	5963	22986
Title length	10.35	11.32	12.17	11.35
Content length	672.33	854.09	598.66	601.35

**Table 2:** Parameter settings table. where *U* is the ranking of all samples, *P* and *Q* are the number of positive and negative samples. The specific parameter settings are shown.

Name	Value
Learning rate	0.0001
Batch size	128
Attention size	64
Query dimension	64
Number of SAM	8
Dropout	0.5
Hidden dimension	128
Kernel	128
Embedding dimension	256
Optimizer	Adam

Figures 7 and 8 reveal that our method’s indicators have the highest overall performance of all the benchmark methods. They have demonstrated an average increase in nDCG@15 and AUC of 5.63% and 6.55%, respectively, proving that the model presented in this article is accurate. This is because our model not only uses GRU to capture the sequential information of users, but also incorporates SAM to better learn the primary interests of users during a session. GRU is used to collect the information that users provide sequentially. In addition, unlike other benchmark methods, the proposed model not only considers the user interaction behavior that occurs within a session, but also the correlation of user interests that occurs between sessions; as a result, the performance of the recommendation has been enhanced even further.

**Figure 9:**
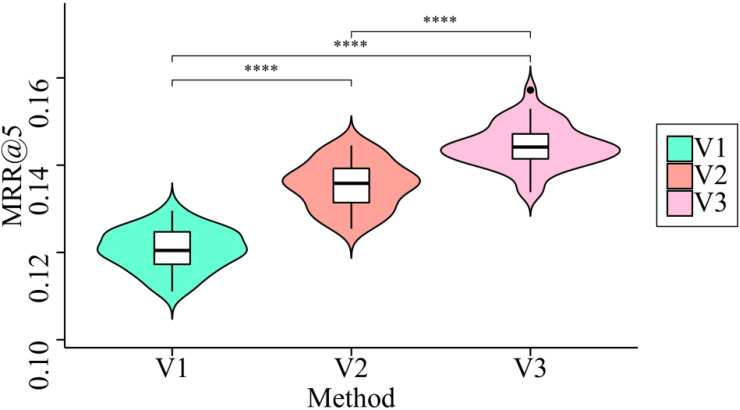
Ablation experiments on three variants of our model. SAM is able to validate the effectiveness of the interest-aware attention layer design because it can determine the proportion of each news item clicked during a session. This figure demonstrates that model performance decreases significantly when SAM is not used to discover user interests during a session.

The following three model iterations are evaluated: (1) The SAM mechanism is not considered for use in Variation 1, as it does not take the primary interests of users during each session into account. (2) In Variation 2, the GRU is not considered for determining the correlation between user interests during the current session and those from previous sessions. (3) Variation 3 considers both the primary interests of users in each session and the relationship between the primary interests of users across sessions. Figure 9 displays the outcomes of experiments conducted on CCX dataset.

SAM is able to validate the effectiveness of the interest-aware attention layer design because it can determine the proportion of each news item clicked during a session. Figure 9 demonstrates that model performance decreases significantly when SAM is not used to discover user interests during a session. When combined, the various weights of have the potential to capture the primary interests of users within a single session. In addition, the session-aware attention layer slightly degrades the experimental results, indicating that it is reasonable to model the correlation of user interests across multiple sessions in order to discover the final user representation. This is possible in order to discover the user’s preferences.

## Conclusion

When mining news features and user features, existing NR models frequently ignore the relationship between browsed news, time series changes, and the importance of various news to users. This results in an incompleteness in the models’ predictions of what users should pay attention to. In the meantime, the models that are currently used provide a deeper level of media coverage. It is unfortunate that there is not enough granular feature mining done in the realm of content. A NR model that is based on a sub-attention news encoder is developed in order to accurately and exhaustively represent users while also extracting finer-grained segment features from news. As part of its deep learning methodology, the model initially pulls a rich feature matrix from the news text using CNN and a sub-attention mechanism. This is the first step in the process. In the second step of the process, the network is built with two student branches, and Conv64 is used to extract the primary features. Granular image data is retrieved by looking at it from the point of view of the image position and channel. Next, a multi-head self-attention mechanism is injected into the user’s news browsing history, and time series prediction is applied to the user’s interests. In the end, but certainly not least, we put the model to the test by contrasting it to other models and evaluating it on both the real-world Chinese dataset as well as the English dataset using metrics such as convergence time, Mean squared error (MSE), Mean squared error (RMSE), and so on. The results of the experiments show that the model that is presented in this article performs better on a wide variety of datasets and converges more quickly in every circumstance. In future work, we will focus on the application of this model to user interaction performance, and verify the validity of the model through examples.

##  Supplemental Information

10.7717/peerj-cs.1246/supp-1 Supplemental Information 1 The code contained in the article This code contains some of the formulas in the article, references, etc.

## Additional Information and Declarations

### Data Availability

The following information was supplied regarding data availability: The code is available in the Supplemental Files. The datasets are available at: - Hugging Face: CCX Dataset: https://www.cisco.com/c/en/us/td/docs/voice_ip_comm/cucm/srnd/collab11/collab11/contactc.html. - GitHub: Microsoft News Dataset: https://github.com/yzh1994414/MIND.git. - MIND: https://msnews.github.io/. - The data used from those datasets are available at Zenodo: Wenting Zhang. (2023). Design of news recommendation model based on sub-attention news encoder [Data set]. Zenodo. https://doi.org/10.5281/zenodo.7536816.
